# Structural Elucidation and Immune-Enhancing Effects of Novel Polysaccharide from* Grifola frondosa*

**DOI:** 10.1155/2019/7528609

**Published:** 2019-04-16

**Authors:** Yu-Ri Seo, Dinesh K. Patel, Woo-Chul Shin, Wan-Sup Sim, Ok-Hwan Lee, Ki-Taek Lim

**Affiliations:** ^1^Department of Biosystems Engineering, Kangwon National University, Chuncheon 24341, Republic of Korea; ^2^The Institute of Forest Science, Kangwon National University, Chuncheon 24341, Republic of Korea; ^3^Department of Food Science and Biotechnology, Kangwon National University, Chuncheon 24341, Republic of Korea

## Abstract

Beta-glucan (*β*-glucan) is a macromolecule structure where glucose unit has bonded through *β*-glycosidic bond at 1 and 3 positions. It is well known as a natural immunomodulator without exhibiting any side effects via enhancing immunity. Mushroom contains a large amount of *β*-glucan and it has anticancerous and antioxidant efficacy. Structure and physical properties of *β*-glucan are highly influenced by the types of mushroom. In particular,* Grifola frondosa* has *β*-1, 3 and *β*-1, 6 bonds in their structure. It has been noted that *β*-glucan content also depends upon the size of mushroom particles. The exact content of *β*-glucan and their immunological activity by a particle size of* G. frondosa* have yet to be fully elucidated. Herein, *β*-glucan contents were analyzed according to the particle size of leaf mushroom followed by cell activation and immunoactivity analysis. The highest *β*-glucan content was observed at a particle size of 20-30 *μ*m (27.65 ± 0.30 w/w). All samples showed ~ 103% cell activation compared to the control and greater cell activity was observed at higher concentration. The significant increase in cytokines secretion was observed in the presence of 20-30 *μ*m particle size of* G. frondosa* compared to the control. This study suggested that 20-30 *μ*m size is the suitable size of* G. frondosa* that can be used as a health supplement and food additive to act as an immune booster, hypotensive agent, and hypoglycemic agent.

## 1. Introduction

Much research on immune enhancement from natural products has been carried out. Notably, there is a growing interest in the efficacy of specific physiologically active substances from natural products, and many studies on natural substances with anticancer, antioxidant, and immunological activity effects have been reported [1]. Mushrooms are one of the most frequently used natural products for immunological activity [2]. Many polysaccharides extracted from mushrooms are effective as anticancer drugs and for immunity control [3]. A lot of edible mushrooms have become attractive as “functional foods” and as source materials for immunomodulators, antitumor agents, antibiotics, and antihypertensive drugs [4].* G*.* frondosa*, one of the outstanding medicinal mushrooms with reported anticancer effects, contains various free amino acids and vitamins B1, B2, C, and D.* G. frondosa* is widely used in Japan, China, and Korea as a traditional food additive due to its texture, delicious taste, and excellent aroma [5]. Unlike other mushrooms, *β*-1, 3 bonds are added to *β*-1, 6 bonds in* G*.* frondosa* [6]. Furthermore, *β*-glucan extracted from* G*.* frondosa* has been reported to enhance antitumor activity and bone marrow toxicity by strengthening the immune system [7]. The key ingredient in improving the immune system by affecting cellular immune recovery is known as *β*-glucan in edible mushrooms [8]. It is a valuable dietary fibre and is a polysaccharide of D‐glucose monomers linked by *β*-glycosidic bonds [9]. The capacity of *β*-glucan to activate immune effector response varies with its structure and particle size. It has also been reported that the size of *β*-glucan particles controls the production of specific cytokines [10]. As interest in *β*-glucan increased, various studies into solvent extraction [11], pH condition [12], and air classification [13] were conducted to improve the content of *β*-glucan. In particular, a mushroom extract prepared from superfine particles has been reported to be useful for immunity enhancement because the activity of lymphocytes was effectively activated in nonclinical experiments [14].* G*.* frondosa* induces enhanced production of cytokines such as interleukin-1, interleukin-6, and tumor necrosis factor *α* [15]. Besides, it has been reported that D-Fraction extracted from* G*.* frondosa* activates interleukin-12 production and also for the T helper-1 cytokine interferon-*γ* and the T helper-2 cytokines interleukin-4 and interleukin-10, thereby eliciting antitumor activity [16]. Studies on the effect of various particle sizes of* G*.* frondosa* on immunity have continued, but few have investigated the evaporation effect of *β*-glucan in microparticulated* G*.* frondosa*. In this study, the content of *β*-glucan was analyzed according to the particle size of* G. frondosa* (10-20, 20-30, and 30-40 *μ*m, respectively). The optimized microsize should be examined and compared to the control sample. Pulverizing* G*.* frondosa* using an air classifying mill could be an effective way to concentrate *β*-glucan content.

## 2. Materials and Methods

### 2.1. Preparation of Materials

The mushrooms (*G*.* frondosa*, Republic of Korea) were treated by an air classifying mill (ACM-AL, Daega, Republic of Korea) that had a pulverizer and a separator. Mushroom samples were pulverized after passing an impeller rotating at high speed in the air classifying milling machine, and a classifier analysis software (Mastersizer S v3.10, Malvern, UK) was used to classify particle size by centrifugal force and drag force. Three fractions labeled as G1, G2, and G3 (10-20, 20-30, and 30-40 *μ*m, respectively) were obtained through air classification. In the process, the feed mass was kept constant at 13 kg, and the rotating speed was set at 3,600 g.

### 2.2. *β*-Glucan Analysis

The quantitative analysis of *β*-glucan was performed using a *β*-glucan kit (Megazyme Inc., Chicago) [17]. The content of *β*-glucan was calculated from the difference between total glucan (*β*-glucan and *α*-glucan) and *α*-glucan. To measure total glucan, 2.0 mL of 12 M sulfuric acid was added to each of the three samples and left for 2 h. Over this period, 10 mL of water was added to each sample and boiled in a water bath at 100°C for 5 min. The samples were then cooled at room temperature. Each sample was subsequently transferred to a 100 mL volumetric flask and 6 mL of 10M potassium hydroxide (KOH) was added. The samples adjust the pH 5 using 200 mM sodium acetate buffer. The samples were centrifuged at 1,500 g for 10 min. The obtained supernatants were transferred into a test tube, and 0.1 mL mixture solution (exo-1, 3-*β*-glucanase/*β*-glucosidase in 200 mM sodium acetate buffer) was added. The samples were incubated at 40°C for 60 min and 3.0 mL of GOPOD Reagent was added. Absorbance was measured at 510 nm. To measure *α*-glucan, 100 mg of each sample was added to a tube with 2 mL of 2 M KOH and stirred for 20 min in an ice bath. Then, 8 mL of 1.2 M sodium acetate buffer (pH 3.8) and 0.2 mL of amyloglucosidase/invertase were added. The samples were incubated at 40°C for 30 min. Next, 0.1 mL of the sodium acetate buffer and 3.0 mL of GOPOD reagent were added before incubation at 40°C for 20 min. Absorbance was measured at 510 nm.

### 2.3. Isolation and Structural Characterization of *β*-Glucan

Isolation of *β*-glucan from* G*.* frondosa *was done as described by Chakraborty et al. [18]. In brief, washing of* G*.* frondosa* particles (20-30 *μ*m) was done with water followed by the ethanol and dried it in an air oven at 45°C for 48 h. After this, extraction of *β*-glucan was performed by boiling the dried particles in 4% NaOH solution for 30 min and kept it overnight at 4°C followed by centrifugation at 4000 rpm at 8°C for 1 h. The supernatant was separated and precipitated with 1:10 (v/v) ethanol and kept it for 48 h at 4°C. The precipitate was collected by centrifugation and repeated wash with ethanol and acetone. The obtained material was extensively dialyzed with cellulose bag to remove the small molecules as well as alkali. The precipitate was collected by centrifugation and freeze-dried. The structural properties of the extracted material were characterized by ^1^H-NMR measurement (400 MHz, JNM-ECZ400S/L1) in Me_2_SO-D_2_O (6:1) and matrix-assisted laser deposition/ionization (time-of-flight), MALDI-TOF mass spectrometry (Bruker Autoflex speed TOF/TOF) as described earlier [19].

### 2.4. Cytotoxicity Test

For the study of cytotoxicity, cell viability experiment was performed with human mesenchymal stem cells (hMSCs). The hMSCs isolated from bone (Korean Cell Line Bank, Republic of Korea) were cultured with proliferative medium (90% DMEM, 10% FBS, and 1% antibiotics) in a culture plate. Cell viability was analyzed using WST-1 assay (EZ-Cytox Cell Viability Assay Kit, Daeillab Service Co., Ltd., Republic of Korea). Before adding the samples (G1, G2, and G3), the hMSCs were cultured in 96-well plates for 24 h. The samples were added to each well at different concentrations (10, 50, and 100 *μ*g/ml). The viability of cultured hMSCs at 24 h after sample adding was assayed using EZ-Cytox according to the described procedure. The cell medium with the untreated sample was set as a control. The absorbance was measured at 450 nm (OD450) using the spectrophotometer.

### 2.5. Antibody-Based Cytokine Array System

Human Cytokine Array C kit was purchased from Ray Biotech (AAH-CYT-1-2, Norcross, GA). The experiment was performed according to the manufacturer's instructions. The array membranes could detect 23 different growth factors/cytokines at a time. To investigate the immune effects of G1, G2, and G3 on cells, 2.2 × 10^6^ cells/well were seeded into 100 mm culture plate. Cell medium without samples served as the control. Cells were grown with samples for 14 days. The cells were then starved to exclude the influence of serum cells for 24 h. Cell-free supernatants were obtained and analyzed by the human cytokine arrays. The membranes were treated with a blocking buffer and then treated with a sample at 4°C overnight. The membranes were then washed with wash buffer, and biotinylated antibody cocktail was added. The membranes were incubated at 4°C overnight and then washed, followed by incubation with the HRP-Streptavidin at 4°C overnight. Finally, the membranes were rewashed and developed by incubation with detection buffer for 5 min. Chemiluminescence of the membranes was measured using the Chemidoc XRS system (BR170-8265, Bio-Rad, USA). Relative protein expression was obtained by comparing the signal intensities.

### 2.6. Statistical Analysis

Data are presented as mean ± standard deviation. All statistical analyses were carried out using SPSS Statistics (IBM SPSS Statistics 23, IBM Inc., USA). Statistical significance between control and treatment groups was compared with a one-way analysis of variance (ANOVA). Statistical significance was considered *∗*p < 0.05.

## 3. Results and Discussion

### 3.1. Analysis of *β*-Glucan Content in Different Particle Sizes

**Figure 1:**
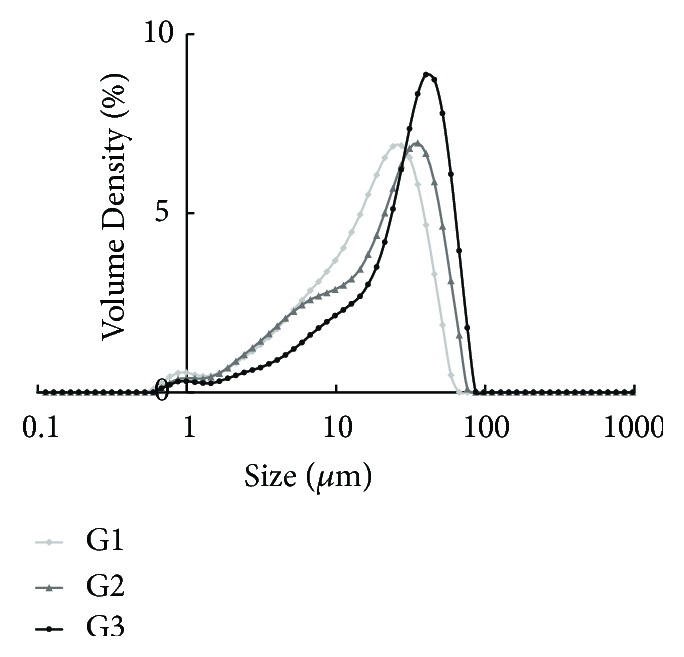
Particle size analysis of the* G. frondosa* powder by air classifying mill.

The three different sizes of* G*.* frondosa* studied in this work were obtained by air classifying mill.  Figure 1 shows the granulometric distribution of the three different particle sizes by Malvern software. The accompanying 10, 50, and 90% values from the cumulative undersize curves are presented in Table 1. The d50 of samples (G1, G2, and G3) was 18.3, 22.8, and 32.7 *μ*m, respectively. Also, high yields were maintained at 97% in the pulverization.

*β*-glucan contents of three different particle sizes in microparticulated* G*.* frondosa* are shown in Table 2. The experiment was repeated three times for each sample. The *β*-glucan contents of G1 and G3 were 18.80 ± 0.78% and 17.67 ± 0.33%, respectively. The highest average *β*-glucan content was found in a particle size of 20-30 *μ*m (G2, 27.65 ± 0.30%). The *β*-glucan content of* G*.* frondosa* was generally reported to be 6.52% [20]. Furthermore, the *β*-glucan contents of* G*.* frondosa* were measured as 25.991% in a recent study [21]. These results indicated that the microsized mushroom could concentrate the *β*-glucan contents. Besides, it was shown that the *β*-glucan content in a particle size of 20-30 *μ*m was more significant than the *β*-glucan content of various mushrooms pulverized to a capacity of 100 *μ*m or less [22].

### 3.2. Structural Analysis of *β*-Glucan

**Figure 2:**
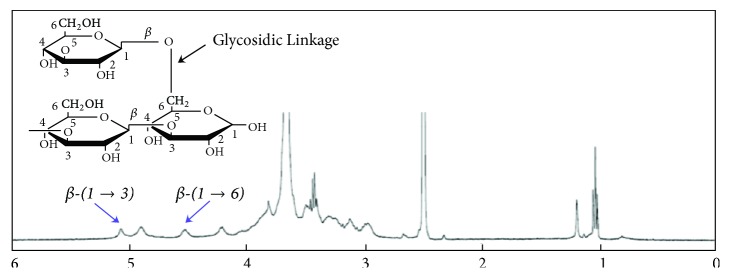
A ^1^H-NMR spectrum of *β*-glucan extracted from* G. frondosa.*

A ^1^H-NMR spectrum of *β*-glucan extracted from* G. frondosa* has been presented in Figure 2. It is well known that anomeric proton signal of the *β*-glucan has occurred in the range of 4-6 ppm depending upon the chemical moieties present in their structure [23]. The appearance of the peaks at 5 and 4.5 ppm in the ^1^H-NMR spectrum confirms the presence of the *β*-(1→3) and *β*-(1→6)-D-glucan linkage in the extracted material, respectively.

**Figure 3:**
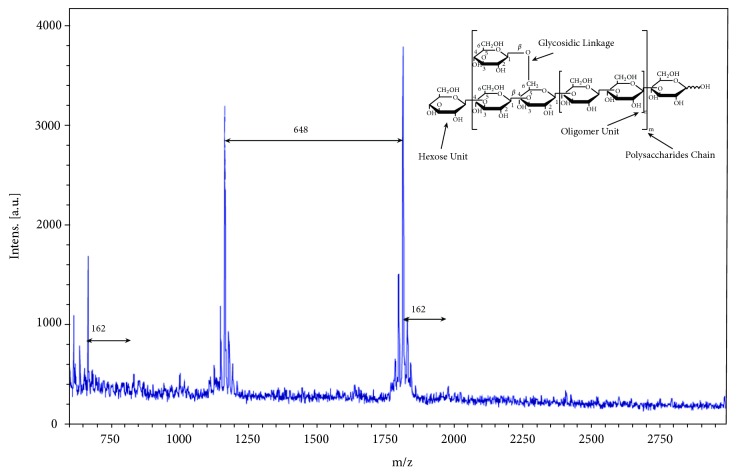
MALDI-TOF mass spectrum of* G. frondosa* glucans using 2, 5-DHB as a matrix. The gap with 162 Da between peaks represents a hexose unit in the extract. Fragmented ions appeared at DP = 4-15 with 667.8, 830.0, 1166.0, 1814.5, 1976.6, and 2406.0 Da, respectively.

MALDI-TOF mass spectrum of *β*-glucan extracted from* G. frondosa* has been presented in Figure 3. MALDI-TOF mass analysis confirmed the presence of the *β*-(1→3) glucan structure in the extract with the molecular mass gap of 162 Da (one hexose unit) as observations have been reported earlier [24, 25]. 2,5-Dihydroxybenzoic acid (2,5-DHB) matrix improved the polysaccharide signals intensity in the MALDI-TOF mass spectrometry. However, it was interesting to note that in-between some peaks molecular gap was 648 Da. This was 4-fold higher than one hexose unit suggesting the presence of oligomer structures in the polymer chain. Mass spectrum of *β*-glucan extracted from the* G. frondosa *indicates that glucans are composed of a mixture of glucose units with molecular weights around 667.8 ~ 2406.8 Da with the degree of polymerization (DP) = 4 ~ 15 which is similar to the earlier reported values of glucan extracted from mushrooms [26]. On the basis of these experiments (NMR and Mass data), we concluded that extracted material has composed of hexose units with *β*-(1→3) and *β*-(1→6)-D-glucan linkage and presumable structure of glucans is given inside the mass spectrum.

### 3.3. Cytotoxicity Test

**Figure 4:**
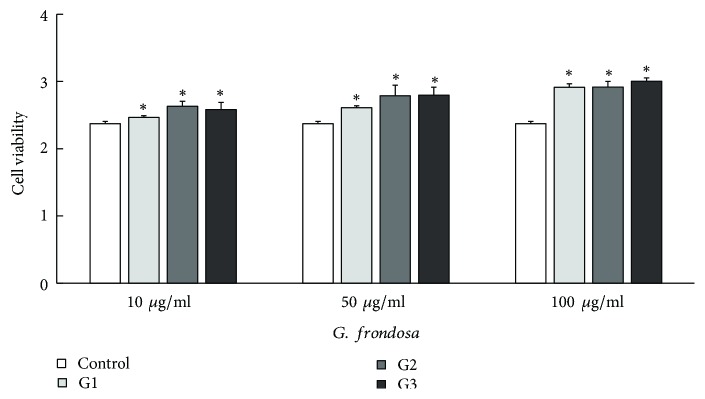
WST-1 assay of hMSCs (bone cell) cultured for different particle sizes of* G. frondosa*. Values are means ± SD for three concentrations per sample. *∗*p < 0.05 compared with the control group.

The in vitro cell culture experiments were performed with hMSCs (bone cell) to investigate the effect of sample concentration and the three different particle sizes on cell proliferation responses. The concentration of samples was set at 10, 50, and 100 *μ*g/ml. The WST-1 assaying of hMSCs cells on an individual has been carried out after incubating bone cells for 2 days (24 h after addition) in a proliferative medium at 37°C. The results of the WST-1 cytotoxicity test for different particle sizes are shown in (Figure 4). From this figure, each of the microparticulated mushroom samples induced a concentration-dependent increase in cell proliferation.* G. frondosa* samples caused a significant increase (*∗*p < 0.05) in cell viability at 50 and 100 *μ*g/ml. The cell viability at 10 *μ*g/ml of G1 was almost the same (p = 0.611) but was higher at the other conditions (50 and 100 *μ*g/ml). In this regard, the proliferation of cells was most accelerated at 100 *μ*g/ml of G3 (increase 126% compared with the control group). These results have confirmed noncytotoxicity for the three different concentrations of G1, G2, and G3.

### 3.4. Protein Expression of Antibody-Based Cytokine Array

**Figure 5:**
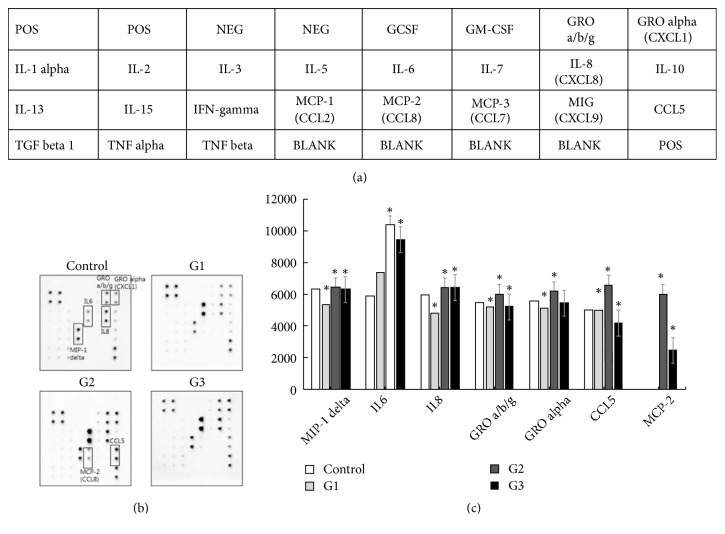
Representative profile of the release of cytokines from hMSCs activated by microparticulated* G. frondosa*. (a) Template showing the location of antibodies for protein spotted onto the human cytokine antibody array kit. (b) Representative expression of various antibodies in the cell-free supernatants with or without* G. frondosa* treated. (c) Relative protein levels detected with the cytokine array. Values are means ± SD for three concentrations per sample. *∗*p < 0.05 compared with the control group.

The cytokine expression profile using a human cytokine protein membrane array is shown in Figure 5. The membrane was exposed only to the culture supernatant of hMSCs. By using the antibody array (Figure 5(a)), the expression of 23 different antibodies in cell-free supernatants with or without* G. frondosa* treatment. The quantitation of cytokine was determined by calculating the intensity of the spot. Seven cytokines were detected as spots on the membranes (IL-6, IL-8, GRO a/b/g, GRO alpha, MCP-2, CCL5, and MIP-1) (Figure 5(b)). The mean for the signal densities for each of the membranes was calculated (Figure 5(c)). Interleukin 6 (IL-6) was upregulated about 126-177% in hMSCs with* G*.* frondosa* treatment. Although interleukin-8 (IL-8) was decreased by the addition of G1, it was increased by about 108% by addition of G2 and G3. Furthermore, the addition of G2 was induced that the three cytokines (chemokine ligand 1 (CXCL1), CC-chemokine ligand 5 (CCL5), and GRO a/b/g) were increased compared to control. When G2 and G3 were added to the cells, they induced monocyte chemotactic protein-2 (MCP-2) production. These results showed the immunomodulation effect on various cytokine levels expressed in the hMSCs with* G. frondosa* treatment. The characteristic features of the cytokines are their functional pleiotropy and redundancy which include interferons, interleukins, stimulating factors, and growth factors, and their regulating activities such as proliferation and differentiation are highly depended upon the nature of the cells involved [27]. These cytokines may exhibit the proinflammatory or anti-inflammatory activities or both, depending upon the target microenvironments [28]. IL-1*β*, TNF*α*, IL-6, IL-15, IL-17, and IL-18 are the most crucial group of cytokines that exhibit inflammatory activities, whereas IL-4, IL-10, and IL-13 are known as anti-inflammatory cytokines. It was noted that the role of inflammatory and anti-inflammatory cytokines in various inflammation concerning their signaling pathways is still ambiguous [29–31].

## 4. Conclusions

*G*.* frondosa* has various physiological activities. *β*-glucan, an essential component of* G*.* frondosa*, affects immune activity. *β*-glucan content in* G*.* frondosa* depends on particle size, raw materials, and extraction conditions. In this study, the particle size of* G*.* frondosa* was microsized to concentrate on *β*-glucan content (10-20, 20-30, and 30-40 *μ*m). Furthermore, the immunostimulatory activity of G.* frondosa* was verified through cell activation ability and cytokine expression. *β*-glucan contents of the microsized* G. frondosa* were higher than generally known *β*-glucan contents for other particle sizes. It was confirmed that the microsized* G. frondosa* effectively concentrated the contents of *β*-glucan. There was also a significant difference in cell proliferation in the treated cells compared to the control. Higher cytokine production was observed in the treated cells than in control. In particular, the addition of G2 tended to increase all seven cytokines (IL-6, IL-8, GRO a/b/g, GRO alpha, MCP-2, CCL5, and MIP-1) expressed. The addition of* G. frondosa* of particle sizes at 20-30, 30-40 *μ*m induced MCP-2 production, but the addition of particle sizes of 10-20 *μ*m did not lead to expression. The expression of cytokines showed substantially higher cytokine production in the treated cells than in control. As a result, the microsized* G. frondosa* promoted cytokine production and is considered to be an excellent medicinal mushroom in terms of enhancing immunity. It is suggested that the increased expression of various cytokines could be useful in the control of immunity through anticancer and antitumor effects.

## Figures and Tables

**Table 1:** Granulometric distribution of *G. frondosa* samples.

Sample	Parameters on Particle Size			Yield (%)
	<d_10_ (*μ*m)	<d_50_ (*μ*m)	<d_90_ (*μ*m)	
G1	3.94	18.3	66.8	97
G2	4.09	22.8	85.8	97
G3	6.67	32.7	90.2	97

**Table 2:** *β*-glucan contents via particle size of *G. frondosa*.

Sample	*β*-glucan (%, w/w)^(1)^	RSD (%)^(2)^
G1	18.80 ± 0.78	4.16
G2	27.65 ± 0.30	1.09
G3	17.67 ± 0.33	1.90

^(1)^All values are expressed as mean ± SD in triplicate.

^(2)^Relative standard deviation.

## Data Availability

The data used to support the findings of this study are available from the corresponding author upon request.
